# Supplementary material for: Genetic enhancement of Trichoderma asperellum biocontrol potentials and carbendazim tolerance for chickpea dry root rot disease management
Source: PLoS One. 2023 Jan 18;18(1):e0280064. doi: 10.1371/journal.pone.0280064 (PMC9847978; doi:10.1371/journal.pone.0280064)
Supplement: S1 Fig — (a) Trichoderma conidia suspensions at108/ml (a); (b) mixing of conidia suspension with talc powder at 1:3 proportion; (c) chickpea seed treatment with Trichoderma formulation and shade drying. (DOCX) [file pone.0280064.s001.docx]

**S1 Fig.** **Preparation of *Trichoderma* formulation and chickpea seed treatment.** (a) *Trichoderma* conidia suspensions at10^8^/ml (a); (b) mixing of conidia suspension with talc powder at 1:3 proportion; (c) chickpea seed treatment with *Trichoderma* formulation and shade drying.

**
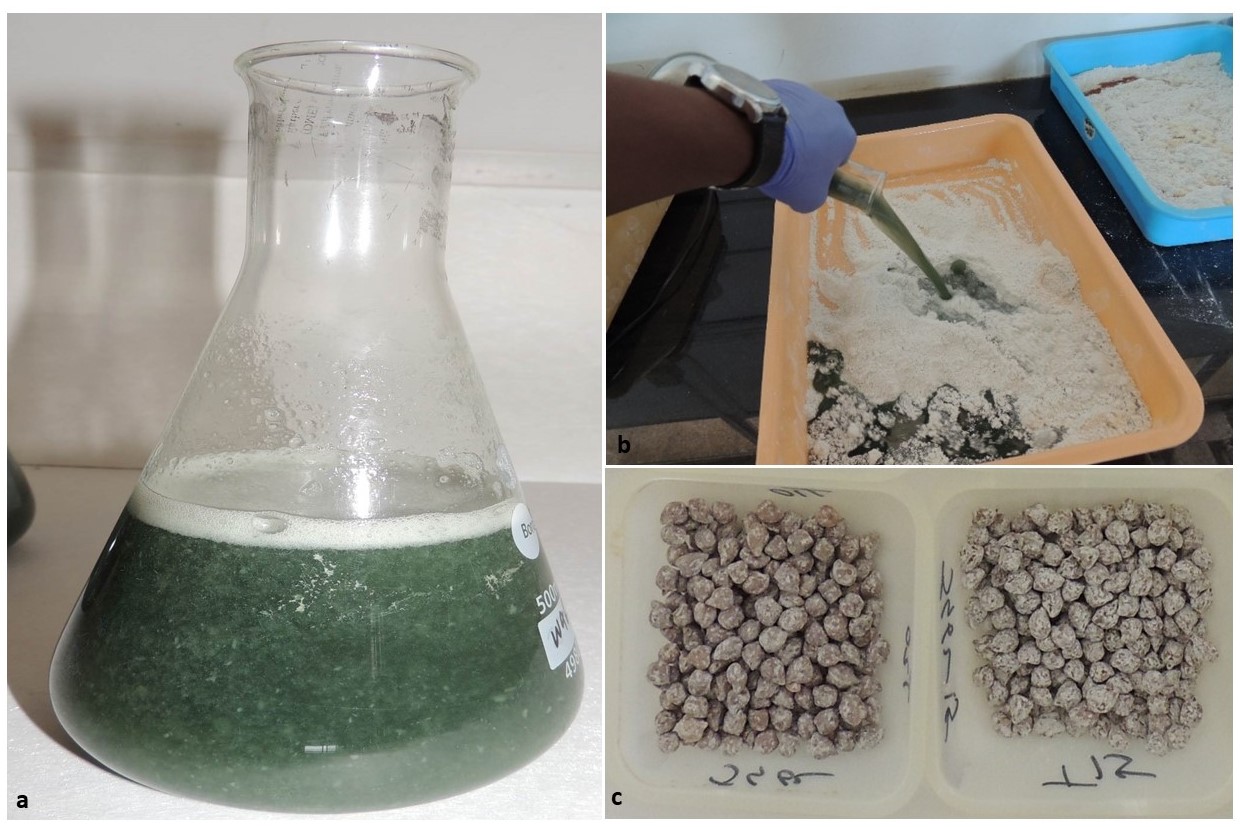
**
