# Supplementary material for: Genetic enhancement of Trichoderma asperellum biocontrol potentials and carbendazim tolerance for chickpea dry root rot disease management
Source: PLoS One. 2023 Jan 18;18(1):e0280064. doi: 10.1371/journal.pone.0280064 (PMC9847978; doi:10.1371/journal.pone.0280064)
Supplement: S2 Fig — (a) Actively growing R. bataticola mycelium in sand-maize meal medium; (b) mixing dry rot inoculum with autoclaved soil and growing on susceptible dry root rot cultivar to confirm the sickness of pots. (DOCX) [file pone.0280064.s002.docx]

**S2 Fig. Preparation of chickpea dry root rot sick pots under glasshouse condition.** (a) Actively growing *R. bataticola* mycelium in sand-maize meal medium; (b) mixing dry rot inoculum with autoclaved soil and growing on susceptible dry root rot cultivar to confirm the sickness of pots.

**
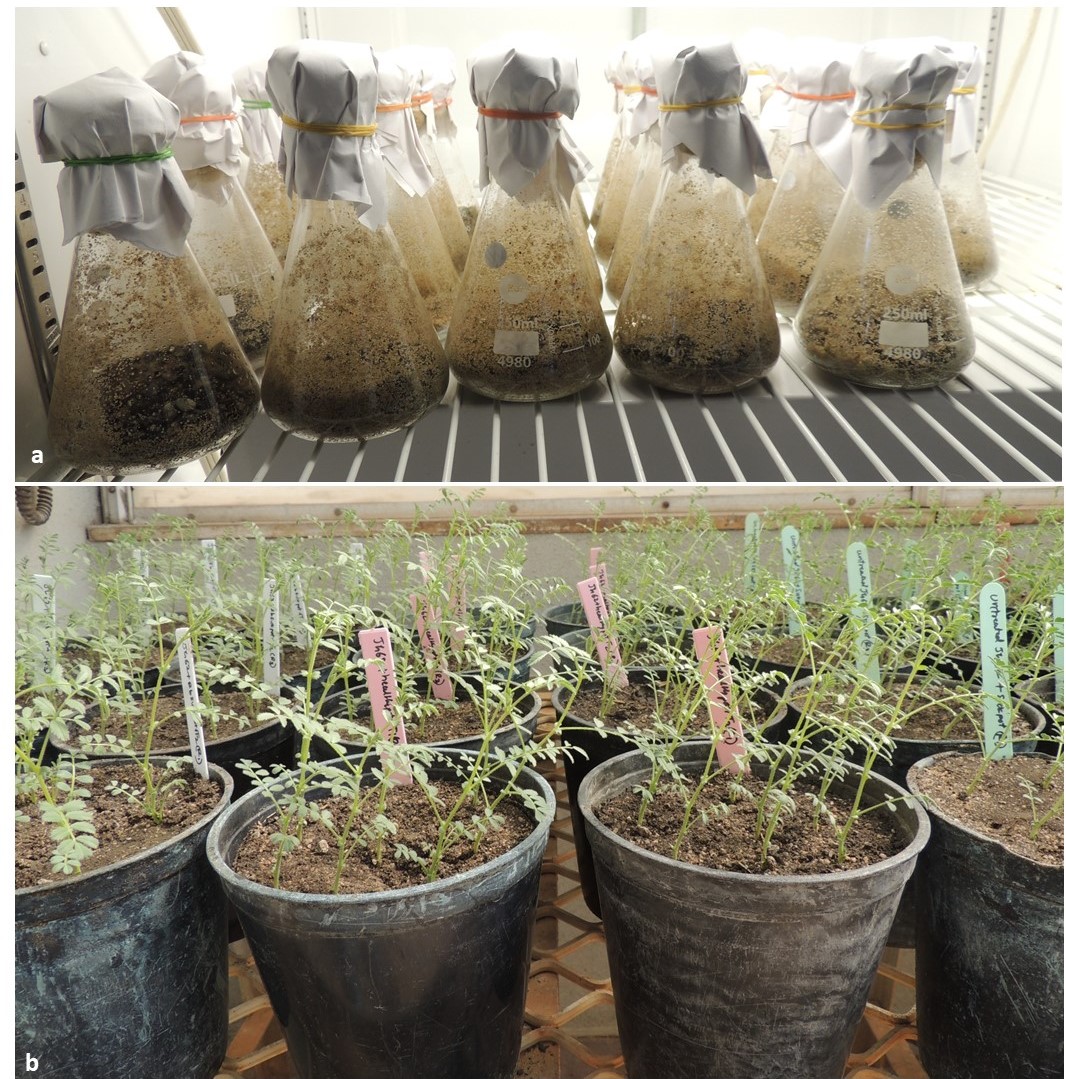
**
