# Supplementary material for: Genetic enhancement of Trichoderma asperellum biocontrol potentials and carbendazim tolerance for chickpea dry root rot disease management
Source: PLoS One. 2023 Jan 18;18(1):e0280064. doi: 10.1371/journal.pone.0280064 (PMC9847978; doi:10.1371/journal.pone.0280064)
Supplement: S3 Fig — Per cent mycelium inhibition of chickpea Fusarium oxysporum f. sp. ciceri, Rhizoctonia bataticola, Botrytis cinerea in dual culture assay and sandwich systems for volatile metabolites effect. (DOCX) [file pone.0280064.s003.docx]

**S3 Fig. Antagonistic and volatile metabolite effect of Trichoderma mutants on chickpea pathogen.** Per cent mycelium inhibition of chickpea *Fusarium oxysporum* f. sp. *ciceri*, *Rhizoctonia bataticola,* *Botrytis cinerea* in dual culture assay and sandwich systems for volatile metabolites effect.

**
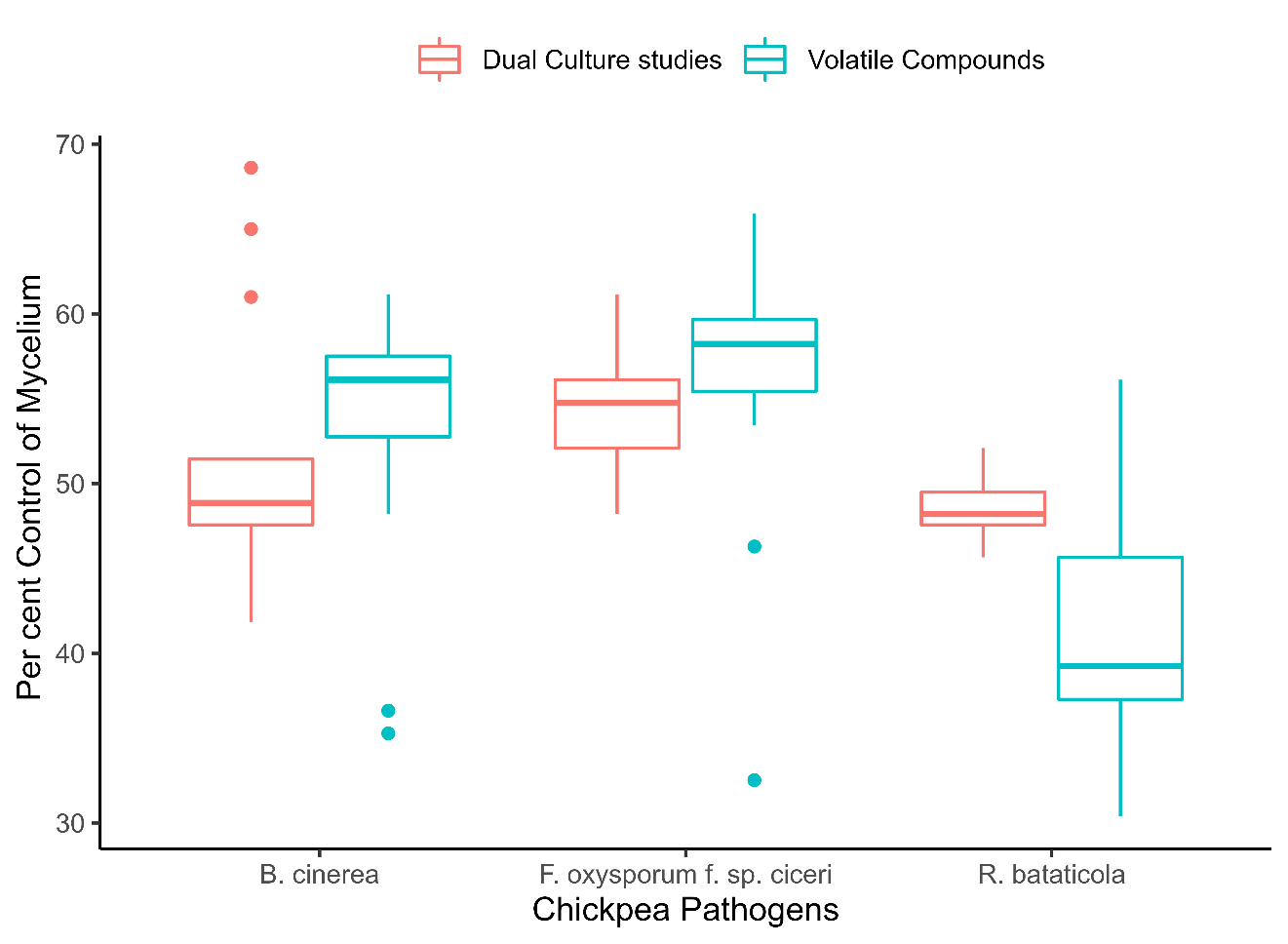
**
