# Supplementary material for: Genetic enhancement of Trichoderma asperellum biocontrol potentials and carbendazim tolerance for chickpea dry root rot disease management
Source: PLoS One. 2023 Jan 18;18(1):e0280064. doi: 10.1371/journal.pone.0280064 (PMC9847978; doi:10.1371/journal.pone.0280064)

**S4 Fig. Conidial germination of N2-2 mutant at different concentrations of carbendazim.** (a) Carbendazim at 0 µg/ml after 24 h; (b & c) carbendazim at 1000 µg/ml after 24 and 48 h; (d & e) carbendazim at 1500 µg/ml after 24 and 48 h.


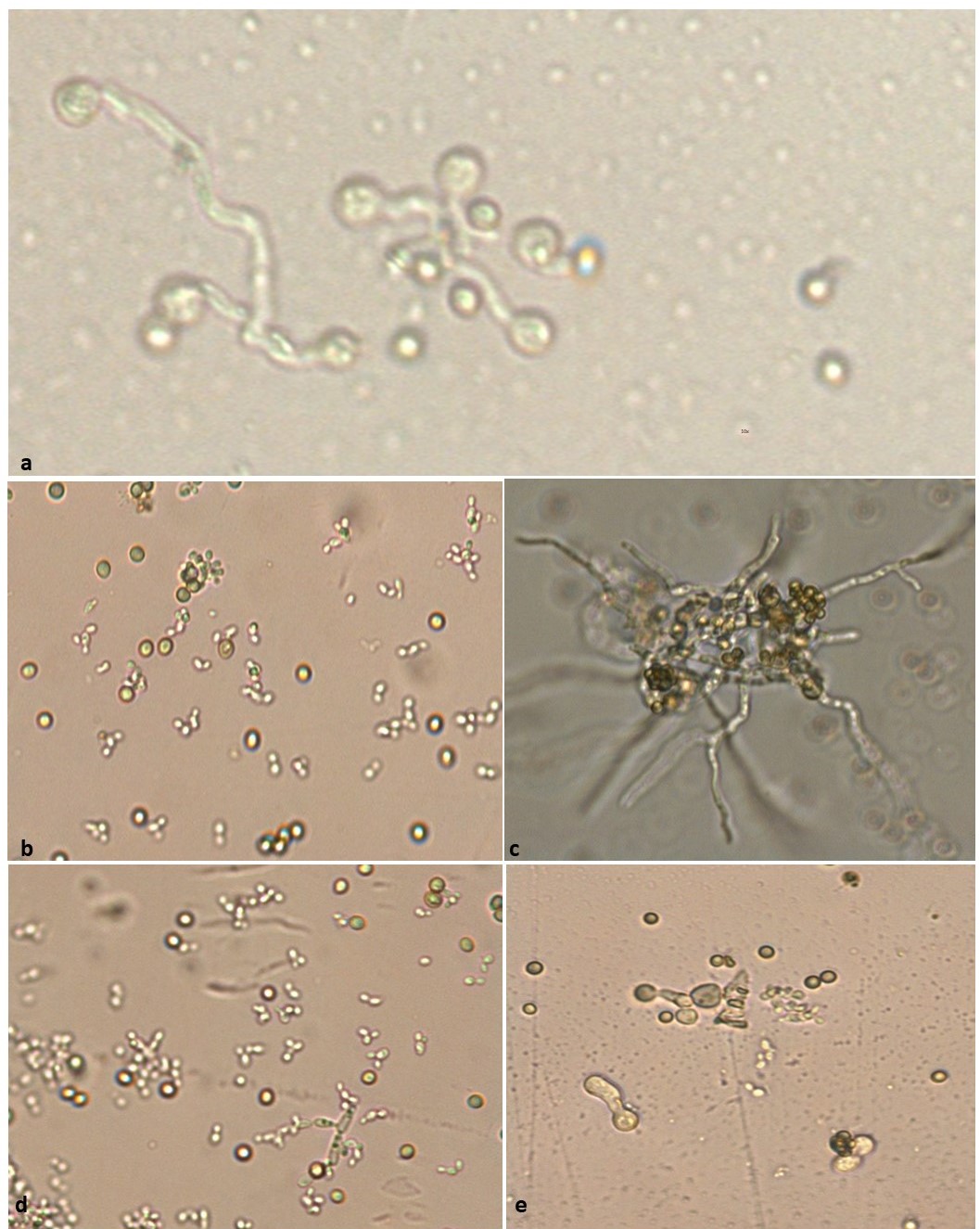

Supplement: S4 Fig — (a) Carbendazim at 0 μg/ml after 24 h; (b & c) carbendazim at 1000 μg/ml after 24 and 48 h; (d & e) carbendazim at 1500 μg/ml after 24 and 48 h. (DOCX) [file pone.0280064.s004.docx]
