# Supplementary material for: Genetic enhancement of Trichoderma asperellum biocontrol potentials and carbendazim tolerance for chickpea dry root rot disease management
Source: PLoS One. 2023 Jan 18;18(1):e0280064. doi: 10.1371/journal.pone.0280064 (PMC9847978; doi:10.1371/journal.pone.0280064)
Supplement: S5 Fig — In the image TaTub2_mt1, TaTub2_mt2 and TaTub_WT -T5 represents the N2, N2-2 and WT strain. (DOCX) [file pone.0280064.s005.docx]

**S5 Fig. Multiple sequence alignment of *Trichoderma asperellum* (N2, N2-2 and WT) *tub2* gene.** In the image TaTub2_mt1, TaTub2_mt2 and TaTub_WT -T5 represents the N2, N2-2 and WT strain.

**
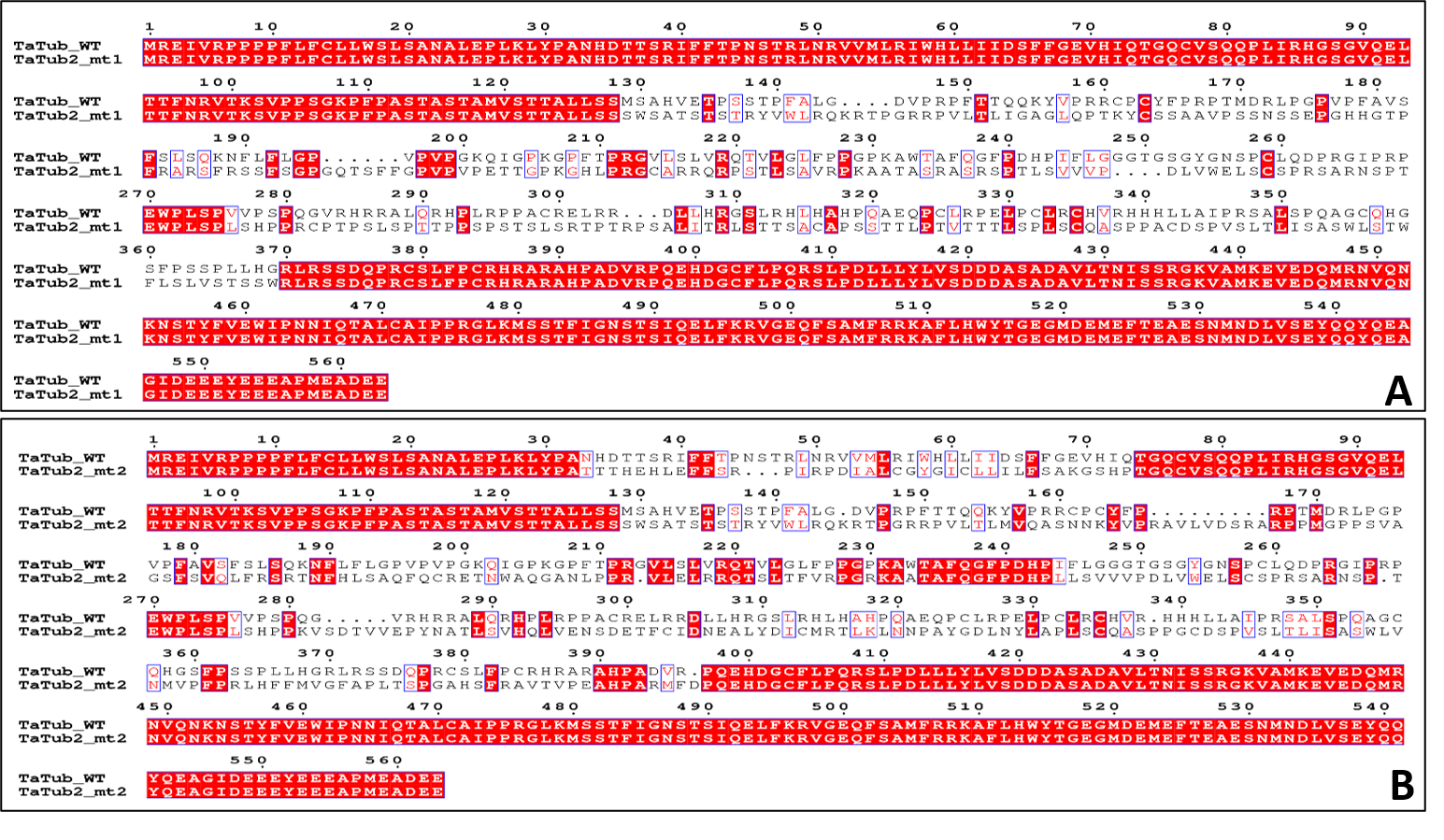
**
