# Supplementary material for: Genetic enhancement of Trichoderma asperellum biocontrol potentials and carbendazim tolerance for chickpea dry root rot disease management
Source: PLoS One. 2023 Jan 18;18(1):e0280064. doi: 10.1371/journal.pone.0280064 (PMC9847978; doi:10.1371/journal.pone.0280064)
Supplement: S1 Table — (DOCX) [file pone.0280064.s006.docx]

**S1 Table. Primer details of *tub1* and *tub2* for amplification of β-tubulin gene.**

| **Gene** | **Forward** | **Reverse** |
| --- | --- | --- |
| Tub1 | CTTTAGCTTCATCCGTCAGTCCGC | GTATGAGCAGTGTCAGTAGTCAAGAC |
| Tub2 | CACAACAGCACCACCTCCAG | GTTAGAACACACTCAGCTCGC |
