# Supplementary material for: Genetic enhancement of Trichoderma asperellum biocontrol potentials and carbendazim tolerance for chickpea dry root rot disease management
Source: PLoS One. 2023 Jan 18;18(1):e0280064. doi: 10.1371/journal.pone.0280064 (PMC9847978; doi:10.1371/journal.pone.0280064)
Supplement: S2 Table — (DOCX) [file pone.0280064.s007.docx]

**S2 Table. PCR reaction and program.**

| **Component** | **1x** |
| --- | --- |
| DNA (100 ng/μl), | 1 μl |
| Buffer A with MgCl2 (10X), | 2.5 μl |
| dNTPs (10 mM) | 0.5 μl |
| Primer (10 pmol/ul) | 1.0 μl |
| *Pfu* DNA Polymerase (3 U/µl) | 0.5 μl |
| Nuclease free water | 19.9 μl |
| Total reaction mixture | 25 μl |
|  | |
| **Program used for PCR** | |
| **Temperature** | **Time** |
| 95 °C | 3 minute |
| 94 °C | 30 seconds |
| 60 °C | 1.5 minute |
| 72 °C | 2 minute |
| 4°C | Hold |
