# Supplementary material for: Genetic enhancement of Trichoderma asperellum biocontrol potentials and carbendazim tolerance for chickpea dry root rot disease management
Source: PLoS One. 2023 Jan 18;18(1):e0280064. doi: 10.1371/journal.pone.0280064 (PMC9847978; doi:10.1371/journal.pone.0280064)
Supplement: S3 Table — (DOCX) [file pone.0280064.s008.docx]

**S3 Table. Integrated management of dry root rot of chickpea disease by carbendazim resistant *Trichoderma* mutants under glasshouse conditions at Annigeri and JG 62 cultivar.**

| **Treatments** | **Percent disease incidence at different days after sowing (DAS) in Annigeri cultivar** | | | | | | | | | | |
| --- | --- | --- | --- | --- | --- | --- | --- | --- | --- | --- | --- |
|  | **20** | **24** | **28** | **32** | **36** | **40** | **44** | **48** | **52** | **56** | **60** |
| T_1_ (sick pot) | 23.81 | 57.14 | 71.43 | 85.71 | 100 | 100 | 100 | 100 | 100 | 100 | 100 |
| T_2_ (WT) | 19.05 | 47.62 | 66.67 | 80.95 | 95.24 | 100 | 100 | 100 | 100 | 100 | 100 |
| T_3_ (N2) | 23.81 | 52.38 | 80.95 | 90.48 | 100 | 100 | 100 | 100 | 100 | 100 | 100 |
| T_4_ (N2-2) | 14.29 | 52.38 | 71.43 | 85.71 | 100 | 100 | 100 | 100 | 100 | 100 | 100 |
| T_5_ (RD of carbendazim) | 0 | 23.81 | 47.62 | 66.67 | 76.19 | 85.71 | 90.48 | 100 | 100 | 1000 | 100 |
| T_6_ (N2+0.5 RD carbendazim)) | 0 | 19.05 | 38.10 | 47.62 | 66.67 | 71.43 | 80.95 | 85.71 | 100 | 100 | 100 |
| T_7_ (N2-2+0.5 RD carbendazim) | 0 | 14.29 | 19.05 | 33.33 | 42.86 | 47.62 | 61.90 | 66.67 | 76.19 | 85.71 | 95.24 |
| T_8_ (healthy pot) | 0 | 0 | 0 | 0 | 0 | 0 | 0 | 0 | 0 | 0 | 0 |
|  | | | | | | | | | | | |
| **Treatments** | **Percent disease incidence at different days after sowing (DAS) in JG 62 cultivar** | | | | | | | | | | |
|  | **20** | **24** | **28** | **32** | **36** | **40** | **44** | **48** | **52** | **56** | **60** |
| T_1_ (sick pot) | 23.81 | 47.62 | 57.14 | 71.43 | 85.71 | 90.48 | 100 | 100 | 100 | 100 | 100 |
| T_2_ (WT) | 14.29 | 38.1 | 57.14 | 76.19 | 80.95 | 95.24 | 100 | 100 | 100 | 100 | 100 |
| T_3_ (N2) | 19.05 | 47.62 | 71.43 | 85.71 | 90.48 | 100 | 100 | 100 | 100 | 100 | 100 |
| T_4_ (N2-2) | 19.05 | 38.1 | 61.9 | 71.43 | 85.71 | 90.48 | 100 | 100 | 100 | 100 | 100 |
| T_5_ (RD of carbendazim) | 0 | 23.81 | 42.86 | 57.14 | 71.43 | 76.19 | 80.95 | 95.24 | 100 | 100 | 100 |
| T_6_ (N2+0.5 RD carbendazim)) | 0 | 14.29 | 28.57 | 42.86 | 57.14 | 71.43 | 76.19 | 90.48 | 90.48 | 95.24 | 100 |
| T_7_ (N2-2+0.5 RD carbendazim) | 0 | 0 | 14.29 | 33.33 | 42.86 | 47.62 | 61.9 | 66.67 | 76.19 | 85.71 | 90.48 |
| T_8_ (healthy pot) | 0 | 0 | 0 | 0 | 0 | 0 | 0 | 0 | 0 | 0 | 0 |
