# Supplementary material for: Genetic enhancement of Trichoderma asperellum biocontrol potentials and carbendazim tolerance for chickpea dry root rot disease management
Source: PLoS One. 2023 Jan 18;18(1):e0280064. doi: 10.1371/journal.pone.0280064 (PMC9847978; doi:10.1371/journal.pone.0280064)
Supplement: S4 Table — Mutations occurring in a) N2 and b) N2-2 analyzed through Provean software. Insertions have been depicted as INS and deletions as DEL. Non-synonymous mutations through substitutions have been depicted as deleterious. (DOCX) [file pone.0280064.s009.docx]

**S4 Table. Mutations occurring in a) N2 and b) N2-2 analyzed through Provean software. Insertions have been depicted as INS and deletions as DEL. Non-synonymous mutations through substitutions have been depicted as deleterious.**

**a)**

| **S. No.** | **Amino acid** | **Position** | **Change to** | **PROVEAN Score** | **Deleterious effect** |
| --- | --- | --- | --- | --- | --- |
| 1 | M | 129 | S | 0.543 | Neutral |
| 2 | S | 130 | W | -0.19 | Neutral |
| 3 | A | 131 | S | -0.058 | Neutral |
| 4 | H | 132 | A | -0.899 | Neutral |
| 5 | V | 133 | T | 0.172 | Neutral |
| 6 | E | 134 | S | 0.317 | Neutral |
| 7 | P | 136 | S | 0.126 | Neutral |
| 8 | S | 137 | T | -0.043 | Neutral |
| 9 | S | 138 | R | -0.222 | Neutral |
| 10 | T | 139 | Y | 0.485 | Neutral |
| 11 | P | 140 | V | 0.331 | Neutral |
| 12 | F | 141 | W | -0.308 | Neutral |
| 13 | A | 142 | L | -0.439 | Neutral |
| 14 | L | 143 | R | 1.612 | Neutral |
| 15 | G | 144 | Q | 0.26 | Neutral |
| 16 | D | 145 | K | 0.071 | Neutral |
| 17 | - | INS | R |  |  |
| 18 | V | 146 | T | -0.084 | Neutral |
| 19 | - | INS | P |  |  |
| 20 | - | INS | G |  |  |
| 21 | P | 147 | R | 0.553 | Neutral |
| 22 | F | 150 | V | 0.098 | Neutral |
| 23 | T | 151 | L | -0.201 | Neutral |
| 24 |  | INS | L |  |  |
| 25 |  | INS | I |  |  |
| 26 | Q | 153 | G | 2.194 | Neutral |
| 27 | Q | 154 | A | 0.384 | Neutral |
| 28 | K | 155 | G | -2.418 | Neutral |
| 29 | Y | 156 | L | -3.325 | Deleterious |
| 30 | V | 157 | Q | -2.541 | Deleterious |
| 31 |  | INS | T |  |  |
| 32 | R | 159 | K | -1.417 | Neutral |
| 33 | R | 160 | Y | -0.446 | Neutral |
| 34 | P | 162 | S | 0.493 | Neutral |
| 35 | C | 163 | S | -0.336 | Neutral |
| 36 | Y | 164 | A | 0.45 | Neutral |
| 37 | F | 165 | A | -0.397 | Neutral |
| 38 | P | 166 | V | -0.401 | Neutral |
| 39 | R | 167 | P | 4.314 | Neutral |
| 40 | P | 168 | S | 0.946 | Neutral |
| 41 | T | 169 | S | -1.735 | Neutral |
| 42 | M | 170 | N | -3.215 | Deleterious |
| 43 | D | 171 | S | -2.915 | Deleterious |
| 44 | R | 172 | S | 1.944 | Neutral |
| 45 | L | 173 | E | 1.065 | Neutral |
| 46 | P | 176 | H | -0.01 | Neutral |
| 47 |  | INS | H |  |  |
| 48 |  | INS | G |  |  |
| 49 |  | INS | T |  |  |
| 50 |  | INS | P |  |  |
| 51 |  | INS | F |  |  |
| 52 |  | INS | R |  |  |
| 53 |  | INS | A |  |  |
| 54 | V | 177 | R | 0.313 | Neutral |
| 55 | P | 178 | S | -0.491 | Neutral |
| 56 | A | 180 | R | -0.857 | Neutral |
| 57 | V | 181 | S | 1.067 | Neutral |
| 58 | L | 185 | DEL |  |  |
| 59 | S | 186 | G | 0.565 | Neutral |
| 60 | Q | 187 | P | 4.157 | Neutral |
| 61 | K | 188 | G | 0.031 | Neutral |
| 62 | N | 189 | Q | -2.629 | Deleterious |
| 63 | F | 190 | T | -2.64 | Deleterious |
| 64 | L | 191 | S | -1.432 | Neutral |
| 65 | L | 193 | F | 0.106 | Neutral |
| 66 | G | 200 | E | -0.203 | Neutral |
| 67 | K | 201 | T | -0.001 | Neutral |
| 68 | Q | 202 | DEL |  |  |
| 69 | I | 203 | T | 1.49 | Neutral |
| 70 | P | 208 | H | 5.826 | Neutral |
| 71 | F | 209 | L | -2.057 | Neutral |
| 72 | T | 210 | DEL |  |  |
| 73 | V | 214 | C | -0.691 | Neutral |
| 74 | L | 215 | A | 1.49 | Neutral |
| 75 | S | 216 | R | -0.509 | Neutral |
| 76 | L | 217 | R | -0.162 | Neutral |
| 77 | V | 218 | Q | -0.191 | Neutral |
| 78 | Q | 220 | P | -0.924 | Neutral |
| 79 | T | 221 | S | 1.367 | Neutral |
| 80 | V | 222 | T | -0.941 | Neutral |
| 81 | G | 224 | S | 0.608 | Neutral |
| 82 | L | 225 | A | -0.246 | Neutral |
| 83 | F | 226 | V | 2.752 | Neutral |
| 84 | P | 227 | R | 4.48 | Neutral |
| 85 | G | 229 | K | 1.703 | Neutral |
| 86 | P | 230 | A | 2.638 | Neutral |
| 87 | K | 231 | A | -0.915 | Neutral |
| 88 | A | 232 | T | -0.05 | Neutral |
| 89 | W | 233 | A | 1.421 | Neutral |
| 90 | T | 234 | S | 0.675 | Neutral |
| 91 | A | 235 | R | -1.779 | Neutral |
| 92 | F | 236 | A | -0.603 | Neutral |
| 93 | Q | 237 | S | -3.237 | Deleterious |
| 94 | G | 238 | R | -5.068 | Deleterious |
| 95 | F | 239 | S | -4.945 | Deleterious |
| 96 | D | 241 | T | 3.138 | Neutral |
| 97 | H | 242 | L | 1.804 | Neutral |
| 98 | P | 243 | S | 3.268 | Neutral |
| 99 | I | 244 | V | 0.013 | Neutral |
| 100 | F | 245 | V | -0.102 | Neutral |
| 101 | L | 246 | V | -1.281 | Neutral |
| 102 | G | 247 | P | -5.225 | Deleterious |
| 103 | G | 248 | DEL |  |  |
| 104 | G | 249 | DEL |  |  |
| 105 | T | 250 | DEL |  |  |
| 106 | G | 251 | DEL |  |  |
| 107 | S | 252 | D | -2.507 | Deleterious |
| 108 | G | 253 | L | -6.669 | Deleterious |
| 109 | Y | 254 | V | 1.257 | Neutral |
| 110 | G | 255 | W | -5.274 | Deleterious |
| 111 | N | 256 | E | -0.609 | Neutral |
| 112 | S | 257 | L | 3.871 | Neutral |
| 113 | P | 258 | S | 0.691 | Neutral |
| 114 | L | 260 | S | 3.804 | Neutral |
| 115 | Q | 261 | P | -1.168 | Neutral |
| 116 | D | 262 | R | 0.236 | Neutral |
| 117 | P | 263 | S | 0.864 | Neutral |
| 118 | R | 264 | A | -0.469 | Neutral |
| 119 | G | 265 | R | 1.769 | Neutral |
| 120 | I | 266 | N | -0.74 | Neutral |
| 121 | P | 267 | S | -0.948 | Neutral |
| 122 | R | 268 | P | 0.466 | Neutral |
| 123 | P | 269 | T | 0.631 | Neutral |
| 124 | V | 276 | L | -2.011 | Neutral |
| 125 | V | 277 | S | -3.153 | Deleterious |
| 126 | P | 278 | H | -5.154 | Deleterious |
| 127 | S | 279 | P | -3.398 | Deleterious |
| 128 | Q | 281 | R | 0.544 | Neutral |
| 129 | G | 282 | C | 0.912 | Neutral |
| 130 | V | 283 | P | 0.273 | Neutral |
| 131 | R | 284 | T | 0.777 | Neutral |
| 132 | H | 285 | P | 3.811 | Neutral |
| 133 | R | 286 | S | 0.726 | Neutral |
| 134 |  | INS | L |  |  |
| 135 |  | INS | S |  |  |
| 136 |  | INS | P |  |  |
| 137 |  | INS | T |  |  |
| 138 |  | INS | T |  |  |
| 139 |  | INS | P |  |  |
| 140 |  | INS | P |  |  |
| 141 |  | INS | S |  |  |
| 142 |  | INS | P |  |  |
| 143 |  | INS | S |  |  |
| 144 | R | 287 | T | 0.716 | Neutral |
| 145 | A | 288 | S | 0.343 | Neutral |
| 146 | Q | 290 | S | 2.71 | Neutral |
| 147 | H | 292 | T | 2.599 | Neutral |
| 148 | L | 294 | T | -1.156 | Neutral |
| 149 | P | 297 | S | 1.526 | Neutral |
| 150 | C | 299 | L | -0.5 | Neutral |
| 151 | R | 300 | DEL |  |  |
| 152 | E | 301 | DEL |  |  |
| 153 | L | 302 | DEL |  |  |
| 154 | R | 303 | DEL |  |  |
| 155 | R | 304 | DEL |  |  |
| 156 | D | 305 | DEL |  |  |
| 157 | L | 306 | DEL |  |  |
| 158 | L | 307 | I | 0.623 | Neutral |
| 159 | H | 308 | T | 1.823 | Neutral |
| 160 | G | 310 | L | 0.082 | Neutral |
| 161 | L | 312 | T | -0.596 | Neutral |
| 162 | R | 313 | T | 2.425 | Neutral |
| 163 | H | 314 | S | -1.565 | Neutral |
| 164 | L | 315 | A | -1.003 | Neutral |
| 165 | H | 316 | C | 3.159 | Neutral |
| 166 | H | 318 | P | -0.161 | Neutral |
| 167 | P | 319 | S | 1.131 | Neutral |
| 168 | Q | 320 | S | 2.2 | Neutral |
| 169 | A | 321 | T | -0.241 | Neutral |
| 170 | E | 322 | T | 0.766 | Neutral |
| 171 | Q | 323 | L | -0.243 | Neutral |
| 172 | C | 325 | T | -1.623 | Neutral |
| 173 | L | 326 | V | -0.712 | Neutral |
| 174 | R | 327 | T | -1.092 | Neutral |
| 175 | P | 328 | T | 2.934 | Neutral |
| 176 | E | 329 | T | 1.074 | Neutral |
| 177 | P | 331 | S | 0.749 | Neutral |
| 178 | C | 332 | P | 0.74 | Neutral |
| 179 | R | 334 | S | 0.697 | Neutral |
| 180 | H | 336 | Q | -0.463 | Neutral |
| 181 | V | 337 | A | 1.063 | Neutral |
| 182 | R | 338 | S | 0.291 | Neutral |
| 183 | H | 339 | P | 0.127 | Neutral |
| 184 | H | 340 | P | -0.394 | Neutral |
| 185 | H | 341 | A | 1.06 | Neutral |
| 186 | L | 342 | C | -2.104 | Neutral |
| 187 | L | 343 | D | 0.512 | Neutral |
| 188 | A | 344 | DEL |  |  |
| 189 | I | 345 | S | 1.019 | Neutral |
| 190 | R | 347 | V | 1.441 | Neutral |
| 191 | A | 349 | L | -1.054 | Neutral |
| 192 | L | 350 | T | -1.434 | Neutral |
| 193 | S | 351 | L | -1.004 | Neutral |
| 194 | P | 352 | I | 0.322 | Neutral |
| 195 | Q | 353 | S | 0.62 | Neutral |
| 196 | G | 355 | S | 0.952 | Neutral |
| 197 | C | 356 | W | -0.916 | Neutral |
| 198 | Q | 357 | L | 2.931 | Neutral |
| 199 | H | 358 | S | -1.37 | Neutral |
| 200 | G | 359 | T | 0.501 | Neutral |
| 201 |  | INS | W |  |  |
| 202 | S | 360 | F | 2.756 | Neutral |
| 203 | F | 361 | L | -0.514 | Neutral |
| 204 | P | 362 | S | -2.997 | Deleterious |
| 205 | S | 363 | L | -0.346 | Neutral |
| 206 | S | 364 | V | 0.654 | Neutral |
| 207 | P | 365 | S | 0.081 | Neutral |
| 208 | L | 366 | T | 0.294 | Neutral |
| 209 | L | 367 | S | 0.648 | Neutral |
| 210 | H | 368 | S | 1.472 | Neutral |
| 211 | G | 369 | W | -0.252 | Neutral |

**b)**

| **S. no.** | **Amino acid** | **Position** | **Change to** | **PROVEAN Score** | **Deleterious effect** |
| --- | --- | --- | --- | --- | --- |
| 1 | N | 33 | T | -0.037 | Neutral |
| 2 | H | 34 | T | -0.044 | Neutral |
| 3 | D | 35 | T | 0.117 | Neutral |
| 4 | T | 36 | H | -0.034 | Neutral |
| 5 | T | 37 | E | -0.058 | Neutral |
| 6 | S | 38 | H | -0.114 | Neutral |
| 7 | R | 39 | L | 0.097 | Neutral |
| 8 | I | 40 | E | -0.014 | Neutral |
| 9 | T | 43 | S | -0.02 | Neutral |
| 10 | P | 44 | R | 0.073 | Neutral |
| 11 | N | 45 | P | 0.001 | Neutral |
| 12 | S | 46 | I | 0.03 | Neutral |
| 13 | T | 47 | R | 0.057 | Neutral |
| 14 | R | 48 | DEL |  |  |
| 15 | L | 49 | DEL |  |  |
| 16 | N | 50 | DEL |  |  |
| 17 | R | 51 | P | -0.055 | Neutral |
| 18 | V | 52 | D | -0.101 | Neutral |
| 19 | V | 53 | I | -0.024 | Neutral |
| 20 | M | 54 | A | 0.1 | Neutral |
| 21 | R | 56 | C | 0.142 | Neutral |
| 22 | I | 57 | G | 0.057 | Neutral |
| 23 | W | 58 | Y | -0.035 | Neutral |
| 24 | H | 59 | G | 0.005 | Neutral |
| 25 | L | 60 | I | -0.007 | Neutral |
| 26 | L | 61 | C | 0.036 | Neutral |
| 27 | I | 62 | L | -0.032 | Neutral |
| 28 | I | 63 | L | -0.062 | Neutral |
| 29 | D | 64 | I | 0.003 | Neutral |
| 30 | S | 65 | L | -0.065 | Neutral |
| 31 | F | 67 | S | 0.238 | Neutral |
| 32 | G | 68 | A | 0.218 | Neutral |
| 33 | E | 69 | K | -0.292 | Neutral |
| 34 | V | 70 | G | -1.767 | Neutral |
| 35 | H | 71 | S | -1.875 | Neutral |
| 36 | I | 72 | H | -1.385 | Neutral |
| 37 | Q | 73 | P | -1.558 | Neutral |
| 38 | M | 129 | S | 0.543 | Neutral |
| 39 | S | 130 | W | -0.19 | Neutral |
| 40 | A | 131 | S | -0.058 | Neutral |
| 41 | H | 132 | A | -0.899 | Neutral |
| 42 | V | 133 | T | 0.172 | Neutral |
| 43 | E | 134 | S | 0.172 | Neutral |
| 44 | P | 136 | S | 0.126 | Neutral |
| 45 | S | 137 | T | -0.043 | Neutral |
| 46 | S | 138 | R | -0.222 | Neutral |
| 47 | T | 139 | Y | 0.485 | Neutral |
| 48 | P | 140 | V | 0.331 | Neutral |
| 49 | F | 141 | W | -0.308 | Neutral |
| 50 | A | 142 | L | -0.439 | Neutral |
| 51 | L | 143 | R | 1.612 | Neutral |
| 52 | G | 144 | Q | 0.26 | Neutral |
| 53 | INS |  | K |  |  |
| 54 | D | 145 | R | 0.885 | Neutral |
| 55 | V | 146 | T | -0.084 | Neutral |
| 56 | INS |  | G |  |  |
| 57 | INS |  | R |  |  |
| 58 | INS |  | V |  |  |
| 59 | INS |  | L |  |  |
| 60 | INS |  | T |  |  |
| 61 | INS |  | L |  |  |
| 62 | INS |  | M |  |  |
| 63 | INS |  | V |  |  |
| 64 | F | 150 | Q | 1.506 | Neutral |
| 65 | T | 151 | A | 0.93 | Neutral |
| 66 | T | 152 | S | -0.029 | Neutral |
| 67 | Q | 153 | N | 0.453 | Neutral |
| 68 | Q | 154 | N | 1.209 | Neutral |
| 69 | R | 160 | A | 2.245 | Neutral |
| 70 | C | 161 | V | 2.275 | Neutral |
| 71 | P | 162 | L | 3.362 | Neutral |
| 72 | C | 163 | V | 2.162 | Neutral |
| 73 | Y | 164 | D | 4.298 | Neutral |
| 74 | INS |  | S |  |  |
| 75 | F | 165 | R | -0.862 | Neutral |
| 76 | P | 166 | A | 0.001 | Neutral |
| 77 | T | 169 | P | -2.539 | Deleterious |
| 78 | D | 171 | G | -3.157 | Deleterious |
| 79 | R | 172 | P | -0.048 | Neutral |
| 80 | L | 173 | P | -1.01 | Neutral |
| 81 | P | 174 | S | 0.918 | Neutral |
| 82 | G | 175 | V | -0.875 | Neutral |
| 83 | P | 176 | A | 1.114 | Neutral |
| 84 | V | 177 | G | 3.237 | Neutral |
| 85 | P | 178 | S | -0.491 | Neutral |
| 86 | A | 180 | S | -0.104 | Neutral |
| 87 | S | 182 | Q | -0.158 | Neutral |
| 88 | F | 183 | L | -1.82 | Neutral |
| 89 | S | 184 | F | 0.109 | Neutral |
| 90 | L | 185 | R | -0.045 | Neutral |
| 91 | Q | 187 | R | -0.31 | Neutral |
| 92 | K | 188 | T | 0.044 | Neutral |
| 93 | L | 191 | H | -1.987 | Neutral |
| 94 | F | 192 | L | -2.403 | Neutral |
| 95 | L | 193 | S | 1.445 | Neutral |
| 96 | G | 194 | A | -0.067 | Neutral |
| 97 | P | 195 | Q | 3.089 | Neutral |
| 98 | V | 196 | F | -0.041 | Neutral |
| 99 | P | 197 | Q | 1.694 | Neutral |
| 100 | V | 198 | C | 0.242 | Neutral |
| 101 | P | 199 | R | 0.073 | Neutral |
| 102 | G | 200 | E | -0.203 | Neutral |
| 103 | K | 201 | DEL |  |  |
| 104 | Q | 202 | T | -0.095 | Neutral |
| 105 | I | 203 | N | 3.191 | Neutral |
| 106 | G | 204 | W | 7.033 | Neutral |
| 107 | P | 205 | A | 2.859 | Neutral |
| 108 | K | 206 | Q | -1.497 | Neutral |
| 109 | P | 208 | A | 0.77 | Neutral |
| 110 | F | 209 | N | -0.625 | Neutral |
| 111 | T | 210 | L | 0.173 | Neutral |
| 112 | R | 212 | P | 0.006 | Neutral |
| 113 | G | 213 | R | -0.564 | Neutral |
| 114 | S | 216 | E | 2.276 | Neutral |
| 115 | V | 218 | R | -0.434 | Neutral |
| 116 | V | 222 | S | -0.281 | Neutral |
| 117 | G | 224 | T | 0.017 | Neutral |
| 118 | L | 225 | F | -1.011 | Neutral |
| 119 | F | 226 | V | 2.752 | Neutral |
| 120 | P | 227 | R | 4.48 | Neutral |
| 121 | P | 230 | R | -0.026 | Neutral |
| 122 | W | 233 | A | 1.421 | Neutral |
| 123 | I | 244 | L | -0.056 | Neutral |
| 124 | F | 245 | L | -0.116 | Neutral |
| 125 | L | 246 | S | -1.252 | Neutral |
| 126 | G | 247 | V | -5.877 | Deleterious |
| 127 | G | 248 | V | -5.908 | Deleterious |
| 128 | G | 249 | V | -6.07 | Deleterious |
| 129 | T | 250 | P | -4.034 | Deleterious |
| 130 | G | 251 | D | -4.82 | Deleterious |
| 131 | S | 252 | L | -3.42 | Deleterious |
| 132 | G | 253 | V | -6.021 | Deleterious |
| 133 | Y | 254 | W | 0.031 | Neutral |
| 134 | G | 255 | E | -5.159 | Deleterious |
| 135 | N | 256 | DEL |  |  |
| 136 | S | 257 | L | 3.871 | Neutral |
| 137 | P | 258 | S | 0.691 | Neutral |
| 139 | L | 260 | S | 3.804 | Neutral |
| 140 | Q | 261 | P | -1.168 | Neutral |
| 141 | D | 262 | R | 0.236 | Neutral |
| 142 | P | 263 | S | 0.864 | Neutral |
| 143 | R | 264 | A | -0.469 | Neutral |
| 144 | G | 265 | R | 1.769 | Neutral |
| 145 | I | 266 | N | -0.74 | Neutral |
| 146 | P | 267 | S | -0.948 | Neutral |
| 147 | R | 268 | P | 0.466 | Neutral |
| 148 | P | 269 | T | 0.631 | Neutral |
| 149 | V | 276 | L | -2.011 | Neutral |
| 150 | V | 277 | S | -3.153 | Deleterious |
| 151 | P | 278 | H | -5.154 | Deleterious |
| 152 | S | 279 | P | -3.398 | Deleterious |
| 153 | Q | 281 | K | 2.533 | Neutral |
| 154 | G | 282 | V | 4.103 | Neutral |
| 155 | V | 283 | S | 3.279 | Neutral |
| 156 | R | 284 | D | 5.131 | Neutral |
| 157 | H | 285 | T | 1.709 | Neutral |
| 158 | R | 286 | V | 0.398 | Neutral |
| 159 | R | 287 | V | 0.856 | Neutral |
| 160 | A | 288 | E | 0.078 | Neutral |
| 161 | L | 289 | P | -1.708 | Neutral |
| 162 | Q | 290 | Y | -1.104 | Neutral |
| 163 | R | 291 | N | 2.432 | Neutral |
| 164 | H | 292 | A | -0.187 | Neutral |
| 165 | P | 293 | DEL |  |  |
| 166 | L | 294 | DEL |  |  |
| 167 | R | 295 | DEL |  |  |
| 168 | P | 296 | DEL |  |  |
| 169 | P | 297 | DEL |  |  |
| 170 | A | 298 | DEL |  |  |
| 171 | C | 299 | DEL |  |  |
| 172 | R | 300 | DEL |  |  |
| 173 | E | 301 | T | 3.083 | Neutral |
| 174 | R | 303 | S | 0.652 | Neutral |
| 175 | R | 304 | V | 1.532 | Neutral |
| 176 | D | 305 | H | 0.501 | Neutral |
| 177 | L | 306 | Q | 0.055 | Neutral |
| 178 | L | 307 | DEL |  |  |
| 179 | H | 308 | DEL |  |  |
| 180 | R | 309 | DEL |  |  |
| 181 | G | 310 | DEL |  |  |
| 182 | S | 311 | DEL |  |  |
| 183 | L | 312 | DEL |  |  |
| 184 | R | 313 | DEL |  |  |
| 185 | H | 314 | DEL |  |  |
| 186 | L | 315 | DEL |  |  |
| 187 | H | 316 | L | 3.311 | Neutral |
| 188 | A | 317 | V | -0.108 | Neutral |
| 189 | H | 318 | E | 0.833 | Neutral |
| 190 | P | 319 | N | 2.344 | Neutral |
| 191 | Q | 320 | S | 2.2 | Neutral |
| 192 | A | 321 | D | 1.438 | Neutral |
| 193 | Q | 323 | T | 2.983 | Neutral |
| 194 | P | 324 | F | 2.833 | Neutral |
| 195 | L | 326 | I | -0.241 | Neutral |
| 196 | R | 327 | D | 1.28 | Neutral |
| 197 | P | 328 | N | 2.293 | Neutral |
| 198 | L | 330 | A | 1.354 | Neutral |
| 199 | P | 331 | L | 3.803 | Neutral |
| 200 | C | 332 | Y | 1.534 | Neutral |
| 201 | L | 333 | D | 1.442 | Neutral |
| 202 | R | 334 | I | -0.341 | Neutral |
| 203 | H | 336 | M | -0.547 | Neutral |
| 204 | V | 337 | R | 0.055 | Neutral |
| 205 | R | 338 | T | -0.267 | Neutral |
| 206 |  | INS | L |  |  |
| 207 |  | INS | K |  |  |
| 208 |  | INS | L |  |  |
| 209 | H | 339 | N | 0.722 | Neutral |
| 210 |  | INS | N |  |  |
| 211 |  | INS | P |  |  |
| 212 |  | INS | A |  |  |
| 213 |  | INS | Y |  |  |
| 214 |  | INS | G |  |  |
| 215 | H | 340 | D | 0.329 | Neutral |
| 216 | H | 341 | L | -0.946 | Neutral |
| 217 | L | 342 | N | -3.201 | Deleterious |
| 218 | L | 343 | Y | -0.1 | Neutral |
| 219 | A | 344 | L | -0.045 | Neutral |
| 220 | I | 345 | A | 0.647 | Neutral |
| 221 | R | 347 | L | 2.177 | Neutral |
| 222 | A | 349 | C | -1.769 | Neutral |
| 223 | L | 350 | Q | -2.41 | Neutral |
| 224 | S | 351 | A | -0.558 | Neutral |
| 225 | P | 352 | S | 1.277 | Neutral |
| 226 | Q | 353 | P | -0.375 | Neutral |
| 227 | A | 354 | P | 0.094 | Neutral |
| 228 | Q | 357 | D | -0.596 | Neutral |
| 229 | H | 358 | S | -1.37 | Neutral |
| 230 | G | 359 | P | 1.13 | Neutral |
| 231 |  | INS | V |  |  |
| 232 |  | INS | S |  |  |
| 233 |  | INS | L |  |  |
| 234 |  | INS | T |  |  |
| 235 |  | INS | L |  |  |
| 236 |  | INS | I |  |  |
| 237 |  | INS | S |  |  |
| 238 |  | INS | A |  |  |
| 239 |  | INS | S |  |  |
| 240 |  | INS | W |  |  |
| 241 |  | INS | L |  |  |
| 242 |  | INS | V |  |  |
| 243 |  | INS | N |  |  |
| 244 |  | INS | M |  |  |
| 245 |  | INS | V |  |  |
| 246 | S | 360 | P | -0.451 | Neutral |
| 247 | S | 363 | R | 0.183 | Neutral |
| 248 | S | 364 | L | -0.57 | Neutral |
| 249 | P | 365 | H | 0.665 | Neutral |
| 250 | L | 366 | F | -0.301 | Neutral |
| 251 | L | 367 | F | 0.355 | Neutral |
| 252 | H | 368 | M | -0.28 | Neutral |
| 253 | G | 369 | V | 1.344 | Neutral |
| 254 | R | 370 | G | 0.537 | Neutral |
| 255 | L | 371 | F | -0.726 | Neutral |
| 256 | R | 372 | A | -0.747 | Neutral |
| 257 | S | 373 | P | -0.584 | Neutral |
| 258 | S | 374 | L | 2.407 | Neutral |
| 259 | D | 375 | T | 0.763 | Neutral |
| 260 | Q | 376 | S | -0.252 | Neutral |
| 261 | R | 378 | G | -1.36 | Neutral |
| 262 | C | 379 | A | 0.816 | Neutral |
| 263 | S | 380 | H | 4.49 | Neutral |
| 264 | L | 381 | S | 0.595 | Neutral |
| 265 | P | 383 | R | -1.563 | Neutral |
| 266 | C | 384 | A | 1.288 | Neutral |
| 267 | R | 385 | V | 1.041 | Neutral |
| 268 | H | 386 | T | 0.229 | Neutral |
| 269 | R | 387 | V | 0.761 | Neutral |
| 270 | A | 388 | P | 2.977 | Neutral |
| 271 | R | 389 | E | 0.05 | Neutral |
| 272 |  | INS | R |  |  |
| 273 | D | 394 | M | 0.732 | Neutral |
| 274 | V | 395 | F | 0.52 | Neutral |
| 275 | R | 396 | D | -0.184 | Neutral |
