# Supplementary material for: Genetic enhancement of Trichoderma asperellum biocontrol potentials and carbendazim tolerance for chickpea dry root rot disease management
Source: PLoS One. 2023 Jan 18;18(1):e0280064. doi: 10.1371/journal.pone.0280064 (PMC9847978; doi:10.1371/journal.pone.0280064)
Supplement: Attachment — Submitted filename: Response reviewers.docx [file pone.0280064.s010.docx]

The authors thank the reviewers for their valuable inputs which has enhanced the quality of our manuscript. The suggested changes have been duly made to the best of our knowledge.

**Reviewer 1**

**Use more recent references**

Thank you for the comment, as suggest new suitable reference were added

**It’s better to know the Elicitors response rather than Beta Tubilin Gene against Mutant strains.**

The authors thank the reviewer for rightfully pointing this out. we are planning a next set of experiments to understand the elicitors responses

**Colony of Trichoderma asperellum show different growth pattern from the growth pattern shown in fig 1 so authenticity of this isolate should be mentioned like Gene bank number etc**

As suggested, GenBank accession number mentioned in the materials and methods sections

**Study done is not similar with abstract. Please elaborate the abstract according to the results.**

Thank you for the comment, as suggested changes are made in the abstract in accord to results

**Reviewer 2**

**The present manuscript is comprehensive and imparts a new insight into biological control of plant diseases. However, there are few revisions as follows.**

**L. 85 (DRR → (DRR)**

Changed (L. 88)

**L. 91 *Trichoderma asperellum* (WT) strain used in the study was obtained from UAS Raichur → Mention the accession number if any**

Added (L. 93)

**L. 125 – 126 Per cent inhibition of mycelial growth was measured → Mention the Formula**

Formula mentioned (L.131-135)

**L. 135 – 136 per cent mycelium inhibition was calculated → Using the formula….**

Changed the sentence (L. 145)

**L. 171 Cultivar Annigeri and JG 62 → Why the authors selected these cultivars for this study, whether they are susceptible for all the three diseases?? If so, indicate that, they are susceptible cultivars for their respective disease.**

Changed the sentence (L.180-181)

**L. 195 – 196 → Mention the PCR conditions for the amplification of *tub1* and *tub2* genes.**

It is mentioned in the S2 table (L. 207)

**Reviewer 3**

L.1 fungicide-resistance →fungicide resistance

L. 36 dry-mycelial →dry mycelial

L. 36 added GenBank accession

L. 93 we don’t have GenBank accession of chickpea pathogens

L. 392 carbendazim-tolerance→ carbendazim tolerance

L. 427 volatile-metabolites→ volatile metabolites

L. 430 fungicide-tolerant→ fungicide tolerant

Table 1 changed

Table 2 changed

Mentioned the abbreviation accept standard once
